# DeepGP: An Integrated Deep Learning Method for Endocrine Disease Gene Prediction Using Omics Data

**DOI:** 10.3389/fcell.2021.700061

**Published:** 2021-07-06

**Authors:** Ningyi Zhang, Haoyan Wang, Chen Xu, Liyuan Zhang, Tianyi Zang

**Affiliations:** ^1^School of Computer Science and Technology, Harbin Institute of Technology, Harbin, China; ^2^Center for Bioinformatics, Harbin Institute of Technology, Harbin, China

**Keywords:** endocrine disease, Graves’ disease, T2DM, PCOS, T1DM, IGF-I, deep learning methods

## Abstract

Endocrinology is the study focusing on hormones and their actions. Hormones are known as chemical messengers, released into the blood, that exert functions through receptors to make an influence in the target cell. The capacity of the mammalian organism to perform as a whole unit is made possible based on two principal control mechanisms, the nervous system and the endocrine system. The endocrine system is essential in regulating growth and development, tissue function, metabolism, and reproductive processes. Endocrine diseases such as diabetes mellitus, Grave’s disease, polycystic ovary syndrome, and insulin-like growth factor I deficiency (IGFI deficiency) are classical endocrine diseases. Endocrine dysfunction is also an increasing factor of morbidity in cancer and other dangerous diseases in humans. Thus, it is essential to understand the diseases from their genetic level in order to recognize more pathogenic genes and make a great effort in understanding the pathologies of endocrine diseases. In this study, we proposed a deep learning method named DeepGP based on graph convolutional network and convolutional neural network for prioritizing susceptible genes of five endocrine diseases. To test the performance of our method, we performed 10-cross-validations on an integrated reported dataset; DeepGP obtained a performance of the area under the curve of ∼83% and area under the precision-recall curve of ∼65%. We found that type 1 diabetes mellitus (T1DM) and type 2 diabetes mellitus (T2DM) share most of their associated genes; therefore, we should pay more attention to the rest of the genes related to T1DM and T2DM, respectively, which could help in understanding the pathogenesis and pathologies of these diseases.

## Introduction

Endocrine diseases fall into broad categories of hormone over- or underproduction, modulate tissue response to hormones, or tumors caused by endocrine tissue (Belfiore and LeRoith, 2018). Hormones synthesized and released by the endocrine glands exert their functions by regulating the biological process of cells. There are several examples of common endocrine diseases: type I/II diabetes mellitus, Graves’ disease (GD), polycystic ovary syndrome (PCOS), and insulin-like growth factor I (IGFI) deficiency, etc. To date, genome-wide association studies (GWAS) have reported numerous gene regions associated with different endocrine diseases. The aim of GWAS analysis is to determine how the combined allele frequency of multiple susceptibility genes can affect autoimmunity and/or disease risk.

Graves’ disease is an organ-specific autoimmune thyroid disease, resulting from excessive secretion of thyroid hormones by thyroid tissue (Dvornikova et al., 2020). The pathogenesis of GD is mediated by the production of antibodies to TSH receptors, which provide increased secretion of thyroid hormones and a rapid growth of the thyroid after stimulation (Smith et al., 2018; Soh and Aw, 2019). Since, GD is a hereditary and polygenic transmission disease (Perricone and Shoenfeld, 2019). It has been identified that associations between CTLA-4, FOXP3, TLR class polymorphism, and a number of pathological conditions develop in GD (Xiao et al., 2015; Fathima et al., 2019).

Diabetes mellitus, such as type 1 diabetes mellitus (TIDM) and type 2 diabetes mellitus (T2DM) are also a typical group of endocrine diseases. But basic pathogenic differences exist in these two types of diabetes mellitus. T1DM is immune mediated while T2DM is mediated by metabolic mechanisms (Eizirik et al., 2020). Glucagon secretion is observed to be reduced in patients with T1DM, with an increasing risk of insulin-induced hypoglycemia, but it is enhanced in T2DM, exacerbating the effects of reduced insulin release and action on glucose of blood levels (Gromada et al., 2018). Recent studies have detected several novel and promising TDM-susceptible genes, such as GCKR, SLC30A8, TLR4, and FTO (Ehrmann et al., 1999; Rotterdam ESHRE/ASRM-Sponsored PCOS Consensus Workshop Group, 2004; Day et al., 2015).

Polycystic ovary syndrome is a common endocrinopathy among women, with symptoms including irregular menstrual cycles, hyperandrogenism and polycystic ovarian morphology (Rotterdam ESHRE/ASRM-Sponsored PCOS Consensus Workshop Group, 2004). It is also accompanied with obesity and insulin resistance, increasing the risk of diabetes, metabolic syndrome, and other cardiovascular diseases (Ehrmann et al., 1999). There are several published causal variants from GWAS studies associated with PCOS (Day et al., 2015; Hayes et al., 2015). Despite the detrimental impact of the disorder on women’s health, the etiology remains poorly understood.

Insulin-like growth factor I production is mainly mediated by growth hormones (GH); both GH and IGF-I have an anabolic effect on skeletal muscle and bone. Despite the effects on growing process in childhood, GH also plays an important role in the regulation of metabolism, body composition, and mood, which persist into adult life (Gazzaruso et al., 2014). However, low serum levels of IGF-I have been detected in patients suffering from chronic liver disease and malnutrition despite normal or elevated GH secretion (Cuneo et al., 1995).

Though there have been numerous susceptible loci identified by GWAS that are associated with endocrine diseases, etiology and pathology is still unclear. Analogous to other complex traits, common susceptibility loci identified by GWAS account for only a small proportion of the genetic heritability of the traits. As GWAS were designed to detect the common allelic variants with a minor allele frequencies of 2 to 5%, the variants occurred less frequently but with greater effect sizes being ignored which may account for the observed deficit in heritability (Manolio et al., 2009). Since SNPs detected by GWAS may not be the real causative regions, the SNPs related to them may be the real causative genes of complex diseases due to the theory of linkage disequilibrium (LD). Therefore, we take the expression level of genes regulated by SNPs into account to reduce the impact of LD. eQTL research plays an important role in prioritizing SNP loci in GWAS susceptible regions (Barral et al., 2012). Previous studies usually investigate susceptible genes of complex diseases based on the regulation function of SNPs on gene expression. MR analysis, for example, is proposed to explain the causative effect on a phenotype of gene expression based on the regulation of genetic variants (Freeman et al., 2013). However, it is rarely available in practice to obtain such a large sample size of different types of data, such as phenotype, genome-wide SNP genotype, and gene expression data, to perform a MR analysis. To overcome this, a SMR method was proposed which integrates summary-level data from independent GWAS with eQTL data to identify disease susceptible genes (Zhu et al., 2016).

Machine learning methods have been widely used in prediction problems, such as support vector machine (SVM), network embedding algorithms (such as node2vec), network diffusion algorithms (such as Laplacian heat diffusion, random walk with restart), etc. Combined with multiple biomolecular features, novel biomarkers, genes, and proteins can be predicted (Chen et al., 2018, 2019; Zhang et al., 2020). Nowadays, deep learning methods have also been utilized in bioinformatics. Liu et al. (2017) applied convolutional neural network (CNN) model to identify cell cycle-regulated genes. Graph convolutional network (GCN) was utilized to predict disease-related metabolites in the study by Zhao et al. (2020). Most machine learning and deep learning methods focused on feature extraction and selection. However, no computational method has been developed to predict the susceptible genes of endocrine diseases based on integrated omics data to eliminate the LD disequilibrium bias.

In this study, we developed “DeepGP,” a method to prioritize susceptible genes of endocrine diseases based on deep learning approaches. First, we obtained curated disease-gene associations of five endocrine diseases from disGeNET database; susceptible regions and expression level data were downloaded from GWAS catalog and GTEx database, respectively. After mapping the genes to susceptible loci based on position information of genes, the feature vector of each gene was composed of two types of features, a phenotype-based feature derived from GWAS dataset and a transcriptome-based feature derived from eQTL data. Disease similarity network can be obtained from our previous work, which can represent disease features. GCN was then utilized to decipher the integrated feature representations of the gene. Finally, the classification of candidate genes was performed by CNN.

## Materials and Methods

### Work Frame

**FIGURE 1:**
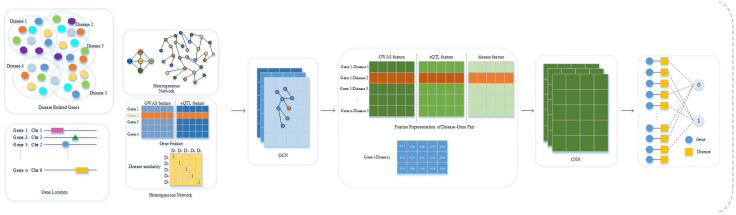
Workflow of DeepGP.

DeepGP contains three main parts, data preprocessing (feature extraction), feature reconstruction based on GCN, and endocrine disease-related gene prediction based on CNN. In the feature extraction process, we obtained endocrine disease-related gene information from DisGeNET (Bauer-Mehren et al., 2010), GWAS Catalog (Buniello et al., 2019), and GTEx Portal databases (Carithers and Moore, 2015). After extracting the features of genes and diseases, we built a heterogeneous network composed of genes and diseases. We then utilized the GCN method to reconstruct the integrated gene features to obtain a more precise feature representation of each gene. In the disease gene prediction part, CNN is used to prioritize the causative genes related to endocrine diseases based on a comprehensive feature representation of disease-gene pairs. The workflow of DeepGP is shown in Figure 1.

### Data Collection and Preprocessing

Genome-wide association studies have identified thousands of genetic variants that are associated with diseases and traits of medical importance in humans. However, the genes detected from the SNPs identified by GWAS which are pathogenic on diseases remain largely unknown due to the complicated LD between SNPs. Intuitively, genes closest to the top associated variants in position are the most likely causative genes. However, there have been studies reporting that causal genes are distinct from the nearest genes (Zhu et al., 2016). Studies have verified that gene expression may be influenced by different genetic variants among genes with different genotypes of the genetic variants, which means the phenotypes can be influenced by genetic variants through regulating the expression of their target genes. Therefore, we used a GCN network embedding method based on two types of omics data to obtain the comprehensive feature vector of each gene.

We first collected endocrine diseases from the “Endocrine diseases” chapter according to the International Classification of Diseases 11th Revision released by the World Health Organization. The major glands of the endocrine system include the pineal gland, pituitary gland, pancreas, ovaries, testes, thyroid gland, parathyroid gland, hypothalamus, and adrenal glands. Considering that some of the endocrine diseases have not been previously widely investigated, we chose five of the endocrine diseases, GD (which is mainly related to thyroid gland), T1DM/T2DM (which is mainly related to pancreas), PCOS (which is mainly related to ovaries), and IGFI deficiency (which is mainly related to pituitary gland). We then collected curated disease genes from the DisGeNET database (Piñero et al., 2016), causative loci from the GWAS database (Buniello et al., 2019), and expression level data from the GTEx database (Carithers and Moore, 2015). In addition, gene–gene interaction network was downloaded from HumanNet v2.0, where the correlation scores between a pair of genes were calculated. We then utilized an R package named biomaRt to obtain detailed information, such as gene location, chromosome number, and start and end position of each gene. BiomaRt can also be used to transform different gene IDs, such as gene symbol, Ensembl ID, and entrez ID, from different databases. After mapping the genes to the susceptible loci identified by GWAS and eQTL, genes with at least one susceptible SNP were kept. Finally, we obtained 7,406 genes, including 4,212 known causal genes and 3,194 candidate genes obtained from HumanNet v2.0.

Therefore, each gene has a feature of 2 × 25D based on GWAS and eQTL summary data according to five diseases. For the phenotype-based feature of each gene, we used the *p*-value of top 5 related SNPs of each disease to indicate the gene feature vector, for genes with less than five related SNPs, the feature vector is filled with 1. Thus, the phenotype-based feature of each gene could be denoted as:

(1) $$G_{S}^{i}=\left[P_{S}^{1}\,P_{S}^{2},\cdots ,P_{S}^{25}\right]\,$$

where $P_{S}^{i}$ denotes the *p*-value of SNPs mapped by gene locations. Transcriptome-based feature of each gene can be extracted by the same method:

(2) $$G_{S}^{T}=\left[P_{T}^{1},P_{T}^{2},\cdots ,P_{T}^{25}\right]$$

where $P_{T}^{i}$ denotes the *p*-value of susceptible loci consistent with that from the phenotype-based feature vector of each gene obtained from eQTL data. Thus, an initial integrated 2 × 25D feature vector of each gene is constructed.

### Feature Reconstruction by GCN

In this section, we introduced a network-embedding algorithm based on GCN in order to present a new representation of gene features. GCN is a graph deep learning method based on node features and network architecture to classify the nodes of a network. Although GCNs have been successfully applied in other domains, to our knowledge, this is the first time that GCN was utilized to represent latent gene features from several omics datasets and network properties, while also being capable of disentangling the underlying molecular mechanisms driving the etiology of endocrine diseases.

Considering a graph *G* = (*V*, *E*, *W*), where *V* denotes the nodes of the network, *E* denotes the edges of the network, and *W* the weight matrix encoding the interacting weight between nodes, which is obtained from HumanNet v2.0 as the gene interactions between gene pairs, the feature matrix of the nodes can be denoted as *X* ∈ *R*^*N*×*F*^. Thus, the eigenvectors of the graph Laplacian L can be denoted as:

(3) $$L=D^{-\frac{1}{2}}\,\tilde{A}\,D^{-\frac{1}{2}}$$

where the adjacency matrix $\tilde{A}=A+I$ has added self-connections since gene nodes should contain both gene interaction and gene itself information and *D* is the degree matrix.

Finally, we can define a propagation rule for each layer:

(4) $$H^{l+1}=\left(L\,H^{l}\,X\,W^{l}\right)$$

(5) $$\text{ReLU}\,\left(x\right)=\left\{ \begin{array}{cc} x, & x>0\\ 0, & x\,\text{≤}\,0 \end{array}\right.$$

where σ denotes a nonlinearity, such as the Rectified Linear Unit activation function. The input of the first layer is *X*, which includes the gene expression feature and gene variation feature of five interested traits, so *H*^0^ = *X*. Therefore, the feature of the gene network could be extracted by formula (4). Since we combined two omics types of data, each gene feature could be represented as a 2 × 25D vector. Thus, we have a feature representation of each gene; the feature can be denoted as: $g_{i}=\left[\begin{array}{c} P_{i,s}^{1},P_{i,s}^{2},P_{i,s}^{3},\cdots ,P_{i,s}^{25}\\ P_{i,T}^{1},P_{i,T}^{2},P_{i,T}^{3},\cdots ,P_{i,T}^{25} \end{array}\right]$.

### Causal Gene Prediction With CNN

After obtaining the best combination of initial feature representation by GCN, we constructed disease features based on disease similarity matrix calculated by the method ImpAESim. Each disease feature can be denoted as a 1 × 5D vector:

(6) $$D_{i}=\left\{S_{i,j}\right\},j=1,2,3,4,5\,$$

*S*_*i,j*_ denotes the similarity between *D*_*i*_ and *D*_*j*_, *S*_*i,j*_ is 1 if *i* = *j*. We then combined the gene feature and disease feature as a 3 × 5D feature of disease–gene pair.

**FIGURE 2:**
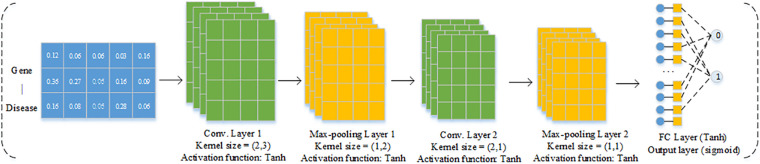
Structure of CNN model.

We then trained a CNN model to predict causal genes based on the gene features derived from GCN. Analogous to other machine learning methods, CNN consists of a training step where the estimation of network parameters from a given training dataset is learned, and the testing step utilized the well-trained network to predict outputs of new testing dataset (Min et al., 2017). Since our feature format of each gene–disease pair is 3 × 5D, which can be regarded as an image with three channels, in this work, the structure of the CNN section is shown in Figure 2. The CNN section includes four parts as follows: convolution layer, max-pooling layer, fully connected layer, and an output layer. Convolution layer is responsible for extracting the subspace features of the input. Max-pooling layer is used for dimension reduction to discard the redundant information. The final fully connected layer connects all the nodes and the output layer applying sigmoid as the activation function to solve the binary classification problem.

The tanh function is the activation function in each convolutional layer.

(7) $$\tanh\left(x\right)=\frac{\sinh x}{\cosh x}=\frac{e^{x}-e^{-x}}{e^{x}+e^{-x}}$$

The sigmoid function is used as the activation function in the output layer.

(8) $$\delta \,\left(x\right)=1/\left(1+e^{-x}\right)\,$$

Since our disease gene prediction can be treated as a binary classification problem, we chose the binary cross-entropy function as the loss function to assess the probability of the output.

(9) $$\mathrm{loss}=-\sum_{i\,\,1}^{n}y_{i}^{{}^{\prime }}\,\log\left(y_{i}\right)+\left(1-y_{i}^{{}^{\prime }}\right)\,\text{log}\,\left(1-y_{i}^{{}^{\prime }}\right)\,$$

(10) $$\frac{\partial \text{loss}}{\partial y}=-\sum_{i=1}^{n}\frac{y_{i}^{{}^{\prime }}}{y_{i}}-\frac{1-y_{i}^{{}^{\prime }}}{1-y_{i}^{{}^{\prime }}}$$

According to formulas (7, 8), loss is 0 as long as $y_{i}^{{}^{\prime }}$ is equal to *y*_*i*_.

As a result, each disease–gene pair was assigned a correlation score with a range of [0, 1], where 1 denotes the pair having the strongest association and 0 means no association.

### Training Steps

According to the 4,212 curated disease-related genes to five endocrine diseases, there are 6,258 positive disease–gene pairs derived from the curated disease–gene associations, and 14,802 (4,212 × 5–6,258) pairs are not reported to be associated; we randomly selected 6,258 pairs to construct the negative samples. However, the sample size of different diseases are extremely unbalanced, as shown in Table 1; the sample size ranges from 28 IGFI–gene pairs to 3,058 T2D–gene pairs due to the insufficiency of studies related to these diseases, which may have a serious negative impact on the classification performance. From the analysis for summary data derived from GWAS and eQTL, T1D, and T2D share as much as 950 genes in total, with 679/1,629 genes merely related to T1D and 2,108/3,058 genes merely related to T2D.

**TABLE 1:** Number of curated disease genes.

**Disease**	**T2D**	**T1D**	**PCOS**	**GD**	**IGFI deficiency**
No. of samples	3,058	1,629	974	568	28
					

After obtaining the new dataset that consisted of 6,258 positive samples and 6,258 negative samples, we conducted a 10-cross-validation on this new dataset to test the performance. First, the dataset is randomly divided into 10 groups, then 10 times of iterations were performed based on nine of 10 groups as training set and one group as test set, which made sure that each group can be used as an independent test set.

## Results

### Performance Evaluation on Predicting Disease–Gene Associations

The area under the curve (AUC) and the area under the precision-recall curve (AUPR) are used to assess DeepGP. The AUC and AUPR of each iteration in 10-cross-validation process are shown in Table 2. As a result, DeepGP achieved a mean AUC of 0.845 and a mean AUPR of 0.833, which have shown better and stable in disease–gene prediction.

**TABLE 2:** AUC and AUPR of DeepGP in 10 times 10 cross-validation.

	**1**	**2**	**3**	**4**	**5**	**6**	**7**	**8**	**9**	**10**	**Average**
AUC	0.832	0.845	0.821	0.854	0.864	0.855	0.831	0.861	0.856	0.831	0.845
AUPR	0.827	0.837	0.816	0.845	0.838	0.825	0.816	0.858	0.842	0.826	0.833

### Comparison Experiments With Classic Methods

We evaluated the performance of DeepGP and classic machine learning methods, such as SVM, random forest (RF), Naïve Bayes, and deep neural network (DNN) for predicting disease–gene associations. To validate the performance of extracting best combination of gene features by GCN, we alternatively only used CNN for feature extraction and prediction. Then, to assess the effect of convolution layers in CNN, we applied a typical deep learning method DNN and two classic machine learning methods SVM and RF. As a contrast, we also only used a SVM model for the classification task. Therefore, we compared the performance of five methods with DeepGP: CNN, GCN-DNN, GCN-SVM, GCN-RF, and SVM.

**FIGURE 3:**
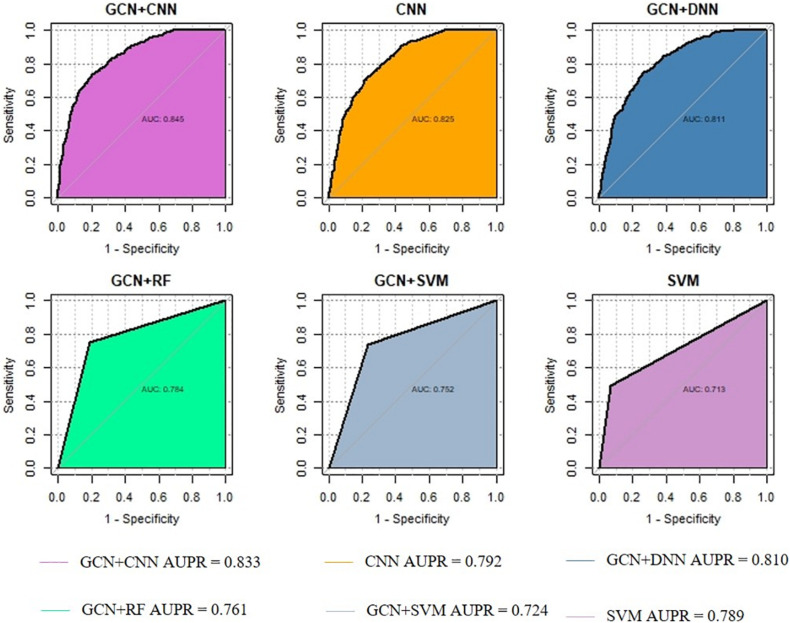
Performance comparison among different methods.

The method of training and testing was performed the same as DeepGP. As shown in Figure 3, comparing with other classic machine learning methods, DeepGP achieves the highest performance according to both AUC and AUPR. CNN and DNN achieved the second and third highest AUROC which infers deep learning methods are better than classic machine learning methods in this disease–gene prediction task; however, it can also be inferred that convolution layers are essential. In addition, the performance was improved after feature encoding by GCN.

**FIGURE 4:**
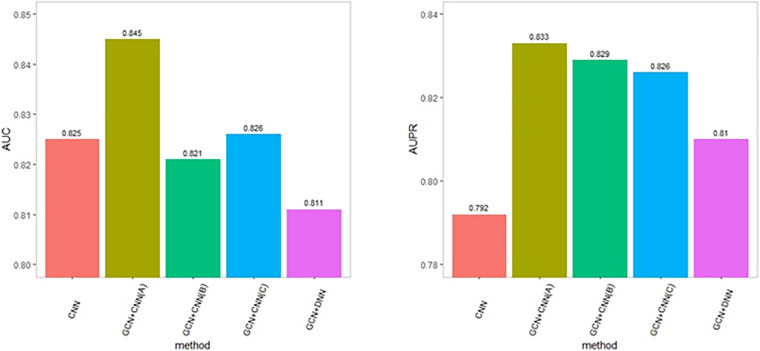
Performance comparison with variational deep learning methods.

It has been shown that the depth of CNN models can affect the classification performance. To assess the influence of the depth of CNN models, we compared DeepGP with a shallower CNN model, denoted as GCN+CNN (B), consisting of one set of one convolution layer and one max-pooling layer. Since the feature dimension of each disease–gene pair is 3 × 5, we also enlarged the kernel size to detect the effect of dimension reduction. The performance is shown in Figure 4. GCN+CNN (A) denotes the structure of DeepGP, GCN+CNN (B) denotes the shallower CNN model, and GCN+CNN (C) denotes the structure of CNN with enlarged kernel size. As a result, DeepGP achieved the best performance comparing with other methods even though the feature dimension of each sample is low. Hence, according to the above results, we conclude that our proposed DeepGP is competitive against other methods.

### Validation of Prediction Results

After verifying the effectiveness of DeepGP based on the comparison experiments. We conducted the disease–gene prediction process among all the unknown disease–gene pairs. We set a threshold of 0.5 as default to screen the scores and identified 7,702 of 14,903 pairs to be true. Among the predicted associations, 971 novel genes were identified to be associated with GD, 3,142 novel genes associated with PCOS, and 2,437 and 1,154 novel genes associated with T1DM and T2DM, respectively. Due to deficiency of studies focused on IGFI deficiency, only 28 of 4,212 were reported as curated genes related to the trait. The highest score of IGFI gene pairs was 0.33, which is under the threshold, and was predicted to be have no associations by DeepGP. For the rest of the diseases, 772 of 3,778 genes were shared.

### Case Study

#### Graves’ Disease

Graves’ disease is mainly mediated by T cells which produce cytokines and chemokines in abnormal amounts. CCL27 is reported to be associated with serum chemokine concentrations detected in GD, which might be a good biomarker for GD (Hiratsuka et al., 2015). CXCL16 have been demonstrated to bind to the unique receptor CXCR6, which is expressed on a subset of multiple types of T cells, and it has been implicated in the pathogenesis of atherosclerosis and GD (Günther et al., 2012). It has been identified that loci at BECN1 can be denoted as a significant extract differentially methylated region for Graves’ orbitopathy under the context of GD (Shi et al., 2019).

#### Polycystic Ovary Syndrome

RBM14 and miR-1307 have been reported to be up-regulated in PCOS patients in the study of Xu et al. (2015) and Che et al. (2020). Besides, CMKLR1, known as chemerin chemokine-like receptor 1, is the receptor of chemerin which is expressed at both mRNA and protein levels in human granulosa cells, and it has been reported to vary in women with PCOS (Bongrani et al., 2019).

#### Diabetes Mellitus

Due to the pathophysiological characteristics and many other potential etiopathogenesis factors T1DM and T2DM share, there are genes linked to both diseases such as *GLIS3* (Mahajan et al., 2014), *EIL2AK3* (also named as *PERK*; Delépine et al., 2000), etc. According to the result obtained from our method, we identified 2,582 genes related to T1D and 1,153 genes related to T2D, with a number of 474 shared genes. We then searched the top-ranked predicted genes related to T1D and T2D. According to the work of Ahmed et al., ADM2 is found to be preferentially up-regulated by bacteroides dorei (BD), which is a bacteria increased significantly at the time of onset of T1D. Enho is an energy homeostasis-associated gene that can produce a regulatory peptides named adropin, which has been identified to be strongly associated with type 1 diabetes in children (Polkowska et al., 2019). *FUT7* gene has been demonstrated to be linked with an antigen termed bile salt-dependent lipase which is reported to be associated with type 1 diabetes (Panicot et al., 1999). UPK3B is regarded as a mesothelial-like cell marker of a major adipocyte progenitor cell subpopulations which may induce adipocyte dysfunction in visceral adipose tissue in type 2 diabetes (Strieder-Barboza et al., 2020). miR-592 is reported to be associated with T2D due to its background of insulin resistance by Song et al. (2019). NELFCD has been identified to be matched with risk haplotypes across five FDM-risk haplotype, which is further identified in a common T2D gene, *ANK1*.

Therefore, we can illustrate that the predicted disease-gene pairs are reliable from the case studies mentioned above; top related genes with diseases are shown in Table 3. The “+” means the gene identified with T1D (T2D) is also related to T2D (T1D) according to the DisGeNET database.

**TABLE 3:** Top 5 related genes with four diseases.

**GD**	**PCOS**	**T1D**	**T2D**
CCL27	RBM14	ADM2 (+)	UPK3B (+)
CXCL16	miR-1307	Enho (+)	miR-592
BECN1	CMKLR1	FUT6 (+)	NELFCD (+)
PROX1	AKT3	FUT7	miR-589 (+)
PTX3	GCGR	ATRNL1 (+)	Linc00641 (+)

## Discussion

In summary, we proposed a disease gene prediction method based on integrated deep learning models. We construct gene features considering both biological process and “linkage disequilibrium” theory. Identifying disease genes merely based on disease-susceptible loci identified by GWAS studies may be inaccurate due to LD. It has been indicated that genetic variants can affect the phenotype by regulating the gene expression level. In this study, eQTL data are also utilized to extract gene features. To fully use the underlying information contained in a gene interaction network, GCN was applied to extract comprehensive gene features based on the constructed gene network. Therefore, our method exploits the predictive power derived from complementary data types and the underlying network simultaneously. Disease features are derived based on disease similarity, which is calculated by the method named ImpAESim. Finally, we combined gene and disease features as a disease–gene pair feature. CNN was then used to classify the disease–gene pairs as a binary classification task.

As a result, DeepGP achieved an average AUC of 0.845 and an average AUPR of 0.833 after a 10 times 10-cross-validation based on the constructed training set, which is superior to other classic machine learning and deep learning methods. We then used the well-trained model to predict the novel disease-gene associations based on the disease–gene pairs which have not been reported to be associated before. We verified the prediction results based on a case study. Most of the top disease-related genes have reported evidences to illustrate that the genes may have associations with the diseases. In addition, we also identified 474 genes shared by T1DM and T2DM which may be helpful in designing therapeutic methods for diabetes mellitus patients. Therefore, the novel disease genes identified by DeepGP provide a strong support for the feasibility of extracting diagnostic markers for future validation and shed light on new strategies for the diagnosis and treatment of endocrine diseases.

## Data Availability Statement

The original contributions presented in the study are included in the article/supplementary material; further inquiries can be directed to the corresponding author/s.

## Author Contributions

NZ did most of the work and wrote the manuscript. HW, CX, and LZ assisted in completing the experiments. TZ proposed the idea of the manuscript. HW made a great contribution in modifying the manuscript. All authors contributed to the article and approved the submitted version.

## Conflict of Interest

The authors declare that the research was conducted in the absence of any commercial or financial relationships that could be construed as a potential conflict of interest.
